# Preparation and Evaluation of Rice Bran‐Modified Urea Formaldehyde as Environmental Friendly Wood Adhesive

**DOI:** 10.1002/gch2.202000044

**Published:** 2021-01-27

**Authors:** Md Nazrul Islam, Md Omar Faruk, Md Nasim Rana, Atanu Kumar Das, Ahsan Habib

**Affiliations:** ^1^ Forestry and Wood Technology Discipline Khulna University Khulna 9208 Bangladesh; ^2^ Department of Forest Biomaterials and Technology Swedish University of Agricultural Sciences Umeå SE‐90183 Sweden; ^3^ Department of Chemistry University of Dhaka Dhaka 1000 Bangladesh

**Keywords:** adhesive properties, defatted rice bran, particleboard properties, rice bran‐based adhesive

## Abstract

In this study, defatted rice bran (RB) is used to prepare an environmentally friendly adhesive through chemical modifications. The RB is mixed with distilled water with ratios of 1:5 and 1:4 to prepare Type A and Type B adhesives, respectively having pH of 6, 8 and 10. Type A adhesive is prepared by treating RB with 1% potassium permanganate and 4% poly(vinyl alcohol), whereas Type B is formulated by adding 17.3% formaldehyde and 5.7% urea to RB. Viscosity, gel time, solid content, shear strength, Fourier transform infrared (FTIR) spectroscopy is carried out, and glass transition temperature (*T*_g_), and activation energy (*E*_a_) are determined to evaluate the performance of the adhesives. *E*_a_ data reveal that adhesives prepared at mild alkaline (pH 8) form long‐chain polymers. Gel time is higher in the fabricated adhesives than that of the commercial urea formaldehyde (UF). FTIR data suggest that functional groups of the raw RB are chemically modified, which enhances the bondability of the adhesives. Shear strength data indicates that bonding strength increases with increasing pH. Similar results are also observed for physical and mechanical properties of fabricated particleboards with the adhesives. The results demonstrate that RB‐based adhesives can be used as a potential alternative to currently used UF‐based resin.

## Graphical abstract

Most of the woodworking synthetic adhesives release volatile‐organic and/or toxic compounds. Thus, there is a pressing demand to produce adhesives having good bondability and that are nontoxic. The defatted rice bran (RB) flour is processed by polycondensation reactions between carbohydrates and crosslinking chemicals for environmentally friendly adhesives. Property evaluation confirms that the RB adhesive has good performance in wood bonding.

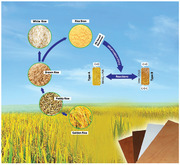

## 1 Introduction

Natural bio‐based raw materials such as starch, protein, and tannin have been used as adhesives for centuries.^[^ ^1^ ^]^ These adhesives lack in bonding quality and water resistance in comparison to the synthetic formaldehyde‐based resin adhesives.^[^ ^2^, ^3^ ^]^ However, the synthetic adhesives emit hazardous gases which are regarded carcinogenic in nature.^[^ ^4^, ^5^ ^]^ As such, researchers nowadays have renewed interest in the study of natural adhesives^[^ ^6^, ^7^ ^]^ and natural bio‐based adhesives.^[^ ^8^, ^9^ ^]^

To diminish toxic pollutants of formaldehyde resins as wood product or composite adhesives, various studies have considered on toxin‐free (nonformaldehyde) adhesives. These are thermosetting or crosslinked‐type polymers, e.g., isocyanate binders and polyvinyl alcohol or various types of natural bio‐adhesives, e.g., natural tannin and proteinaceous or starch‐based adhesives.^[^ ^10^, ^11^, ^12^, ^13^, ^14^ ^]^ Among the natural bio‐based resins, rice (*Oryza sativa*) bran (RB) has not been studied in detail.^[^ ^9^ ^]^ RB is a by‐product of rice milling and produced from brown rice through abrasive milling to produce pure polished rice.^[^ ^15^, ^16^ ^]^ Currently, an estimated 80–85 MT (8–10% of produced rice) of RB is produced per year worldwide.^[^ ^17^ ^]^ The likely composition of RB is 12–15% protein, 15–20% fat, 36% starch, and other inorganic materials.^[^ ^18^, ^19^ ^]^

In the production of new form of adhesives, defatted RB flour may be useful as a raw material. However, few reports have been published related to production of glue from the RB, e.g., thermal and hydraulic sodium treated RB,^[^ ^9^, ^20^ ^]^ fortified RB with 30% polymeric diphenyl diisocyanate^[^ ^21^ ^]^ and with poly(vinyl alcohol) (PVA) at adjusted pH.^[^ ^22^ ^]^ Urea formaldehyde (UF) mixed RB adhesive was prepared with better properties in comparison to the other adhesive system of RB.^[^ ^23^ ^]^ However, the research on the modification or production of RB adhesive is still in its infancy. The production of RB‐based adhesive could increase the economic value of this less important by‐product and provide environmentally friendly products for the wood‐based industry. Thus, the objective of this investigation was to produce and evaluate the properties of the rice bran‐based adhesive for application in wood‐based industries specially to fabricate composites.

## 2 Experimental Section

### 2.1 Materials

RB is locally available in Bangladesh and was collected from Arafat Auto Rice Mill Ltd., Khulna, Bangladesh. The urea‐formaldehyde (UF) resin used for this study was commercial grade UF (pH 8, gel time 2.30 min, and solid content 48%) provided by Akij Particle Board Mills Ltd. (APBML), Manikganj, Bangladesh.

### 2.2 Preparation of Materials

RB was defatted according to a standard protocol reported elsewhere.^[^ ^24^ ^]^ The RB powder was added in hexane solution at a ratio of 1:3 (w/v). The mixture was then stirred at room temperature using a stirrer (Glassco: 700.AG.01, India) at a setting of 300 rpm for an hour. The mixture was then centrifuged by a centrifuge machine (Thermo Scientific Fibertile Carbon Rotors, USA) at 4000 rpm for 10 min. The defatted RB was collected from the centrifuge tube through decanting the supernatant that contained fat into a beaker. The residue was washed twice by hexane and then dried to obtain the defatted RB powder.

### 2.3 Formulation of RB Adhesives

#### 2.3.1 Type A

The defatted RB powder was mixed with distilled water at a ratio of 1:5 (w/v), and the slurry pH was adjusted to 6, 8, and 10 by adding either diluted H_2_SO_4_ or NaOH solution. Then 1% potassium permanganate (KMnO_4_) solution was gradually added to the RB slurry and stirred. After the addition of KMnO_4_ solution, the slurry was heated in a water bath with shaking (Reciprocal Shaking Water Bath, JSSB‐30T, Korea) at 70 °C for 1.5 h. PVA powder was added to the slurry at a ratio of 1:6 (w/w). The PVA powder was divided into two equal portions. The first half was slowly added to the slurry at 70 °C for 30 min. After addition of the PVA, the heater was stopped and kept the slurry for 5 min. Later, the rest portion of the PVA was added. The slurry was then stirred at 300 rpm for 1 h. The mixture was stored in the refrigerator at 4 °C until its usage as adhesive.

#### 2.3.2 Type B

The defatted RB powder was mixed with distilled water at a ratio of 1:4 (w/v). About 2 mL of methanol was poured to the slurry as an antifreezing material. The slurry pH was adjusted to 6, 8, and 10 by adding either diluted H_2_SO_4_ or NaOH solution. The mixture was heated at 90 °C for 1 h. About 6.5% formaldehyde was added dropwise to the slurry with stirring at 120 rpm for 5 min and then heated at 90 °C for 55 min. The slurry was cooled down to reach room temperature. Later, about 5.7% solid urea and 10.8% formaldehyde were added over a period of 10 min at room temperature and 15 min at 75 °C into the solution at 80 rpm. Immediately after adding formaldehyde, 3 g NaOH was added to the solution and heated for another 90 min. It was then ready to use as adhesive and was stored in the refrigerator at 4 °C until further use.

### 2.4 Characterization of the Formulated RB Adhesives

#### 2.4.1 Pretreatment Yield

After pretreatment, the wet RB was dried at room temperature for 12, 24, and 48 h to calculate the pretreated RB yield. The dried RB was milled for 3 min and sieved through a US no. 100 mesh screen. The sieved portion containing particles smaller than 0.15 mm was further used to produce rice bran adhesive. Pretreated RB yield was determined with the following Equation (1)

(1) $$\begin{array}{c} \text{Yield}\left(\%\right)\;\;=\;\;\frac{A_{t}-A_{r}}{A_{t}}\;\;\times \;\;100 \end{array}$$

where *A*_t_ is the total amount of oven‐dried RB, and *A*_r_ is the amount retained on the 100 mesh sieve.^[^ ^20^ ^]^

#### 2.4.2 Gel Time

RB adhesive (25 g) and ammonium chloride (NH_4_Cl) (1 g) were added into a beaker and were fully mixed. The mixtures of 5 g in the beaker were transferred to a test tube. A stirring rod was inserted into the test tube for checking the condition. The test tube together with the stirring rod was placed in a short neck flask with boiling water, and gel time formation of the adhesive was determined with a stopwatch. Each value was obtained from the average of three tests.

#### 2.4.3 Solid Content

The adhesive samples of 5 g were placed on a piece of aluminum foil, which were dried to constant weight. The aluminum foils with preloaded adhesive were put in a vacuum drying oven (Vacuum Oven, OV‐11, Korea). The temperature of the oven was increased to 103 ± 2 °C and maintained under vacuum for 1 h. Then, the aluminum foils together with the dry adhesive samples were transferred to a desiccator and cooled about 20 min. The solid content (*R*) of the adhesive was determined from the following formula (2)

(2) $$\begin{array}{c} R\;\;=\;\;\frac{m_{2}-m_{0}}{m_{1}-m_{0}} \end{array}$$

in which *R*, *m*_o_, *m*_1_, and *m*_2_ are solid content, weight of aluminum foil, weight of aluminum foil with wet adhesive sample, and weight of aluminum foil with dry adhesive sample, respectively.^[^ ^25^ ^]^

#### 2.4.4 Viscosity

The viscosity of the prepared adhesive was determined at room temperature using a spindle viscometer (Sheen VM1‐R, UK) with L no. 4 spindle. Viscosity was measured at 100 and 200 rpm immediately after vigorous stirring, and each value was obtained from the average of three tests.

#### 2.4.5 Glass Transition Temperature (*T*_g_) Analysis

The *T*_g_ was measured by using differential scanning calorimetry (DSC) (LABSys evo, Setaram Instrumentation, France) according to ASTM E1356 procedure under nitrogen atmosphere in a temperature range from room temperature (25 °C) to 600 °C with a heating rate of 10 °C min^−1^.

#### 2.4.6 Determination of Activation Energy (*E*_a_)

To estimate the activation energy in physical or chemical processes, data obtained at several non‐isothermal curing kinetic tests performed using DSC at constant heating rates, i.e., 5, 10, and 15 °C min^−1^ (constants during each test, different among tests) under N_2_ atmosphere. According to the Kissinger equation, the values of activation energy can be obtained from the slope by plotting ln(β/*T*_P_^2^) versus (1/*T*_P_)^[^ ^26^ ^]^

(3) $$\begin{array}{c} E_{a}\;\;=\;\;-R\;\;\frac{d\left(\ln\left(\frac{\beta }{T_{p}{}^{2}}\right)\;\right)}{d\;\left(\frac{1}{T_{p}}\right)} \end{array}$$

where *E*_a_ is the activation energy (kJ mol^–1^), *R* is the universal gas constant (8.315 J mol^–1^ K^–1^), β is the constant heating rate, and *T*_p_ is the maximum peak temperature obtained from plots of heat flow versus temperature.^[^ ^27^ ^]^

#### 2.4.7 Fourier Transform Infrared Analysis of the Two Types of Adhesives

Infrared (IR) spectra of the prepared adhesive samples were performed using a Fourier transform infrared (FTIR) spectrometer (PerkinElmer, USA). Each of the spectra was recorded in wave number with a range of 600–4000 cm^–1^ using attenuated total reflection (ATR) method coupled with a diamond crystal.

#### 2.4.8 Shear Strength Test

The shear strengths of the RB adhesives were measured by using the Universal Testing Machine (UTM) (SHIMADJU, 50 KN, Japan) followed by ASTM D 905 block shear specimens (shear area = 50 × 40 mm^2^) and EN 205 single lap joint (shear area = 20 × 20 mm^2^) methods as the test of adhesives for bonding wood products. Temperature, press time length, and pressure were maintained at 25 °C, 5 min, and 5 MPa, respectively, throughout the experiment.

### 2.5 Manufacturing of Particleboard Panels

Particleboard panels were manufactured in the laboratory of Forestry and Wood Technology Discipline of Khulna University, Bangladesh. Wood particles of 0.5–1.0 mm were dried in the oven at 103 ± 2 °C for 24 h to reach a constant weight. Then 100 g of dried particles were manually mixed with 12 g of each type of RB adhesive and UF (control). These mixtures were placed separately in a mold (wooden box) to form a mat. A hot press machine (Carver, USA) was used to press each type of mat at 180 °C temperature with a pressure of 5 MPa for 10 min. The target density of the fabricated particleboard panels was 0.60 g cm^−3^. The fabricated panels were trimmed, and the dimension of each type of particleboard was 330 × 330 × 5 mm. The panels were then conditioned at room temperature for 1 day before testing the properties. There were five replications for each type of panels for this study.

#### 2.5.1 Physical and Mechanical Properties Tests

The density of each type of board was measured according to the ASTM standard (D2395, 2017). The dimensional stability of the boards was checked by measuring thickness swelling (TS) and water absorption (WA), and the mechanical properties of the boards were analyzed by determining modulus of elasticity (MOE), modulus of rupture (MOR), tensile strength and hardness of the boards according to the ASTM standard (D1037,1999). The sample size for TS and WA was 50 × 50 × 5 mm. The samples were soaked in water at room temperature for 2 and 24 h to determine the TS and WA. The samples were weighed before and after soaking in water. Mechanical properties, three‐point bending properties using center‐point loading, were determined by UTM (SHIMADJU, 50 KN, Japan). The sample size for mechanical properties was 150 × 50 × 5 mm. There were five replications for analyzing both types of physical and mechanical properties for this study.

#### 2.5.2 Formaldehyde Emission Analysis

The formaldehyde emission analysis from the fabricated particleboards was done following the standard method EN 717‐2. The sample dimension was 20 × 30 × 5 mm, and edge was sealed with self‐adhesive aluminum tape. The sample was placed in a 4 L chamber maintaining temperature, relative humidity, and airflow of 60 ± 0.5 °C, 3%, and 60 ± 3 L h^−1^, respectively. The samples were kept in the chamber for 4 h, and the emitted formaldehyde was absorbed in water. Then, it was determined by photometric acetylacetone (AcAc) method. This is according to Hantzsch reaction^[^ ^28^ ^]^ method where formaldehyde reacts with ammonium ions obtained from ammonium acetate and AcAc to produce 3,5‐diacetyl‐1,4‐dihydrolutidine (DDL) having yellow color. A spectrophotometer was used to read the color of DDL at 412 nm for determining the formaldehyde concentration.

### 2.6 Data Analysis

Data analysis was performed using “RStudio” version 1.1.463.^[^ ^29^ ^]^ The descriptive statistics (means, SD, SEs, etc.) were calculated using the “psych” package;^[^ ^30^ ^]^ normality and homogeneity were tested with the “car” package.^[^ ^31^ ^]^ Appropriate transformation was applied to yield normal distributions for all interested traits. An ANOVA model was also applied with the “car” package at 5% significance level. All graphs were made with the “ggplot2” package^[^ ^32^ ^]^ and “Origin 8.”^[^ ^33^ ^]^

## 3 Results and Discussion

### 3.1 Pretreatment Yield

The effect of drying time on the RB yield at room temperature is shown in **Table** **1**. Results showed that RB yields decreased from 71.2 to 59.9% with the increasing of drying time for 48 h drying at room temperature. However, Pan et al.^[^ ^20^ ^]^ reported that RB yield was decreased from 60 to 35% for 48 h drying period. Oven‐dried samples provided lower yield with highly dense and compact adhesives compared to those having 48 h drying period at room temperature.^[^ ^20^ ^]^ Short drying period provided high RB yield with low dense adhesive, whereas long period provided high dense adhesive with low RB yield. The low yield RB adhesives were found to be harder than those prepared from short drying period. This could be attributed to long drying period, which facilitated the removal of moisture, loosely bound water, and possibly some volatile organic compounds from the adhesives. The compactness of highly dense RB adhesives could be because of the increase of cohesiveness of the chemical constituents of the adhesives through formation of some sort of bonding. Short drying period provided higher yield with low dense RB adhesives; however, they showed weaker bonding ability.

**Table 1:** RB yields on different drying periods at room temperature

Drying periods [h]	RB yield [%]
12	71.8 ± 0.7
24	66.7 ± 0.53
48	59.9 ± 0.21

### 3.2 Gel Time and Solid Content

The gel time of synthetic adhesives was shorter than that of natural adhesives. This is because the main raw materials of synthetic adhesives were almost pure that facilitated fast chemical bonding, thereby resulting in shorter gel time (2.15–3.00 min).^[^ ^34^ ^]^ On the other hand, natural raw materials collected from various sources are usually subjected to direct and/or mild chemical treatment for the preparation of natural adhesives. Thus, the availability of the active sites/functional groups of the natural raw materials is supposed to be limited in the formation of chemical bonding and take longer time for gel formation. The effect of gel time on solidification rate of the RB adhesives is shown in **Table** **2**. Results showed that the gel times for Type A were 5.47, 5.41, and 5.26 min at pH 6, 8, and 10, respectively, and for Type B were 5.48, 5.39, and 4.57 min at pH 6, 8, and 10, respectively. However, the gel time for UF was only 2.30 min at pH 8.^[^ ^34^ ^]^ As seen in Table 2, the gel time for solidification decreased with increasing the pH in case of chemical treatment. The change in gel time for both types of adhesives for changing pH from 6 to 8 was negligible, but a considerable change was observed, especially for Type B adhesive for the pH change from 8 to 10. These results suggested that the active sites and/or functional groups were regenerated by alkali treatment. The generated active sites and/or functional groups took part in the formation of chemical bonding thus resulting in fast gel formation, alternatively achieving shorter gel time. However, the effects of chemical treatment on the percentage of solid content were negligible for both the types. Fast gel formation alternatively shorter gel time is required for practical application of natural materials in preparation of cost‐effective and environmentally friendly adhesives in fabrication of composites.

**Table 2:** Effect of gel time on solidification rate of the prepared rice bran (RB) adhesives

Adhesive properties	Adhesives						
	Type A			Type B			UF
	pH 6	pH 8	pH 10	pH 6	pH 8	pH 10	pH 8
Gel time [min]	5.47 ± 0.02	5.41 ± 0.03	5.26 ± 0.03	5.48 ± 0.02	5.39 ± 0.01	4.57 ± 0.02	2.30 ± 0.01
Solid content [%]	39.4 ± 0.13	39.8 ± 0.18	40.3 ± 0.1	37.1 ± 0.08	39.9 ± 0.09	40.5 ± 0.07	48 ± 0.5

### 3.3 Viscosity

**Figure 1:**
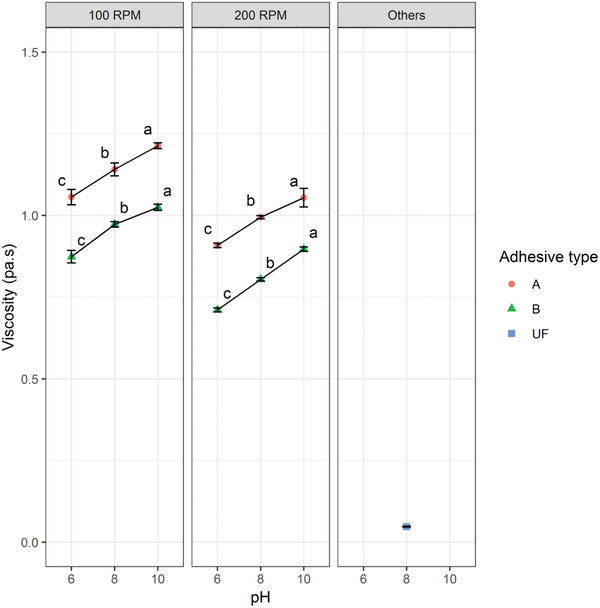
Effect of pH and chemical treatment methods on viscosity of the prepared RB adhesives measured at 100 and 200 rpm and others (Ford viscosity cup method as used by the industry).

The effect of pH and chemical treatment methods on the viscosity of the prepared RB adhesives measured at 100 and 200 rpm is shown in **Figure** **1**. As seen from Figure 1, for 100 rpm, the viscosities for Types A and B were increased significantly (ANOVA, *F*_2.33_ = 2441.60, *P* < 0.05) from 1.05 to 1.21 Pa s and from 0.87 to 1.02 Pa s and for 200 rpm, from 0.90 to 1.05 Pa s and from 0.71 to 0.89 Pa s for pH change from 6 to 10, respectively. The increasing trend of viscosities of the prepared RB adhesives might arise owing to the gelatinization of starch molecules and denaturation of proteins at alkaline pH, i.e., 8 and 10. The increase of the solidification rate of the RB adhesives in alkaline pH confirmed the increasing trend of viscosities of the RB adhesives. Pan et al.^[^ ^20^ ^]^ also reported that the viscosity of RB increased with the increase of pH in the alkaline region. Leach et al.^[^ ^35^ ^]^ reported that starch took part in hydration reaction at alkaline pH, and gelatin formation occurred which mainly enhanced the viscosity. Medium pH significantly affected the structure of proteins and other biomolecules so that the alkaline pH (pH 8–10) denatured the proteins of the rice bran (RB) resulting in increasing of viscosity of the RB adhesives at higher pH (alkaline).^[^ ^36^, ^37^ ^]^ Interestingly, relatively higher viscosities were observed for Type A adhesives than those for the Type B. However, adhesives with low viscosity are desired for easy handling and applying in industrial production process.^[^ ^20^ ^]^ In this study, the observed viscosities of the RB adhesives were quite high compared to the UF adhesive; however, Type B showed relatively lower value than that of the Type A. Pan et al.^[^ ^20^ ^]^ also reported higher value of viscosities for the alkali‐treated RB adhesives. Mixture of RB with UF provided the viscosity lies in between the RB and UF viscosities.^[^ ^23^ ^]^

### 3.4 Glass Transition Temperature (*T*_g_)

**Figure 2:**
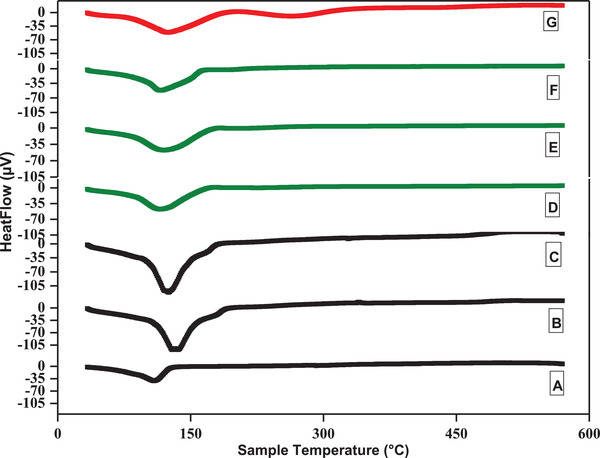
Glass transition temperature (*T*_g_) of A) Type A pH 6, B) Type A pH 8, C) Type A pH 10, D) Type B pH 6, E) Type B pH 8, F) Type A pH 10 and G) urea formaldehyde, respectively.

Determination of the glass transition temperature (*T*_g_) of polymeric materials is of paramount importance. As the polymeric materials change their physical states with temperature, *T*_g_ provides information about the nature of the polymeric materials at the given temperature. *T*_g_ of the prepared RB adhesives was measured by using a DSC. **Figure** **2** shows the effect of pH on the *T*_g_ and heat capacity (*C*_p_) of the RB adhesives of Type A and B along with UF adhesive. It has been observed that the thermograms exhibit endothermic type, and both the *T*_g_ and *C*_p_ have increased with the increment of pH. The *T*_g_ values at pH 6, 8, and 10 for Type A were 117, 138, and 138 °C, respectively, and *C*_p_ values were −53.72, −210, and −150 µV, respectively. For Type B, the *T*_g_ values were 134, 142, and 153 °C, and *C*_p_ values were −39.23, −71.81, and −99.02 µV, for pH 6, 8, and 10, respectively. As mentioned in Section 3.4, viscosity of the RB adhesives also increases with the increase of pH. At low pH, possibly short‐chain polymeric molecules were mainly formed, whereas longer chain polymeric molecules predominated at higher pH, i.e., 8 and 10. Both the short‐ and long‐chain polymeric molecules of the RB adhesives form in amorphous materials, but short‐chain molecular network is probably relatively weakly bonded than the longer chain molecular network.^[^ ^38^ ^]^ By introducing temperature, the adhesive molecules absorbed enough heat at some point and the amorphous structure changed to flexible and rubbery structure. Under these circumstances, the polymeric adhesive molecules moved freely around each other resulting in transition from rigid to flexible and rubbery state. Hence, strongly bonded adhesives prepared at higher pH value (e.g., 8 or 10) showed relatively higher *T*_g_ values.

### 3.5 Activation Energy (*E*_a_)

**Figure 3:**
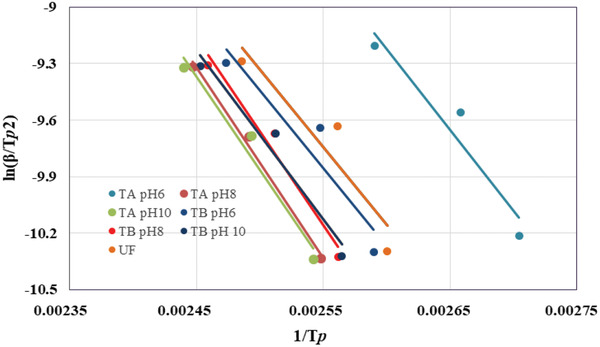
Determination of activation energy using Kissinger method for different types of adhesives.

Activation energy (*E*_a)_ is defined as the minimum amount of extra energy alternatively energy barrier required by reacting species to get converted into products. The *E*_a_ values for the curing kinetics of RB adhesives were calculated from the slopes of the plots of the ln(β/*T*_P_^2^) versus (1/*T*_P_) through applying the non‐isothermal Kissinger method (**Figure** **3**).^[^ ^26^ ^]^ As seen in Figure 3, the ln(β/*T*_P_^2^) versus (1/*T*_P_) showed straight lines, and the *E*_a_ values were calculated from the slopes of the straight lines. The values of *E*_a_/RT, *E*_a_, and linear range correlation coefficient (*R* ^2^) derived by using the Kissinger equation for different types of RB and UF adhesives are given in **Table** **3**. Results showed that *E*_a_ increased as a function of pH, and the values were 71, 84, and 82 kJ mol^−1^ for pH 6, 8, and 10, respectively, for Type A and that for Type B were 69, 83, and 75 kJ mol^−1^, respectively (Table 3). These results can be ascribed to the increase of viscosity of the RB adhesives because of denaturation of proteins of RB adhesives at alkaline pH. Denaturation of proteins involves the disruption and/or destruction of secondary and tertiary structures rather than hydrolysis and converts into long‐chain amino acid polymeric molecules. On the other hand, at acidic pH (6.0), the protein molecules take part in partial hydrolysis, thus resulting in decreasing in viscosity of the RB adhesives as well as in formation of shorter chain amino acids polymeric molecules. The long‐chain polymeric molecules gain strong intermolecular interactions such as van der Waals force, hydrogen bonding, dipole–dipole interaction, and so on, whereas short‐chain polymeric molecules might have weak covalent bonding. Therefore, the low *E*_a_ value of the prepared RB adhesives at acidic pH (6.0) is logical.

**Table 3:** Values of activation energy obtained using Kissinger method

Adhesive type	*T*_p_ [°C]			−(*E*_a_/RT)	*E*_a_ [kJ mol^−1^]	*R* ^2^
	5	10	15			
TA pH 6	96.63	103	112.9	8598	71	0.9208
TA pH 8	119.3	128.21	135.49	10 143	84	0.9924
TA pH 10	120.2	127.91	136.71	9875	82	0.964
TB pH6	113	119.42	131.14	8220	69	0.9000
TB pH8	117.23	125	133.46	9925	83	0.9693
TB pH10	116.76	124.9	134.53	8965	75	0.9596
UF	111.39	117.29	129.11	8243	69	0.8767

The strong covalent bonding in the long‐chain polymeric molecules slows down the curing reaction ultimately increasing the energy of activation (*E*_a_) of the RB adhesives at alkaline pH such as 8.0 and 10.0.^[^ ^39^ ^]^ Moreover, formation of discrete domains in the RB adhesives at alkaline pH, 8.0 and 10.0 act as diluents that decrease the reactivity of the long‐chain polymeric molecules, thereby resulting in increasing the value of *E*_a_.^[^ ^27^ ^]^ Singh et al.^[^ ^27^ ^]^ reported that the value of the activation energy, *E*_a_, of epoxy resin was increased from 45 to 60 kJ mol^−1^ by addition of aliphatic reactive diluents (RD) into the epoxy resin.^[^ ^27^ ^]^ The presence of RD in the epoxy resin acts as catalysts for deactivation of the reactive epoxy polymeric molecules. The slight decrease in *E*_a_ values at pH 10.0 can be ascribed to blocking the active sites for formation of hydrogen bonding of the RB adhesive molecules by sodium ions. Consequently, the force of intermolecular interaction of the RB adhesive molecules reduces that enhances their reactivity. This is the reason to get relatively low values of *E*_a_ for both types of RB adhesives at pH 10.0. However, Vertuccio et al.^[^ ^40^ ^]^ and Singh et al.^[^ ^27^ ^]^ have reported that curing schedule, resin to hardener ratio, curing process, and so on can also increase the values of *E*_a_.^[^ ^27^, ^40^ ^]^ In this study, chemical and pH treatments may play role in variation of processing and curing schedule as well. As seen in Table 3, the observed *E*_a_ values for the RB adhesives were higher than that of UF adhesive. The linear range correlation coefficient (*R* ^2^) was found within a range from 0.89 to 0.99 that confirms the analytical validation of the obtained values of the activation energy (*E*_a_) through constructing the ln(β/*T*_P_^2^) versus (1/*T*_P_) curves.

### 3.6 FTIR Analysis

**Figure 4:**
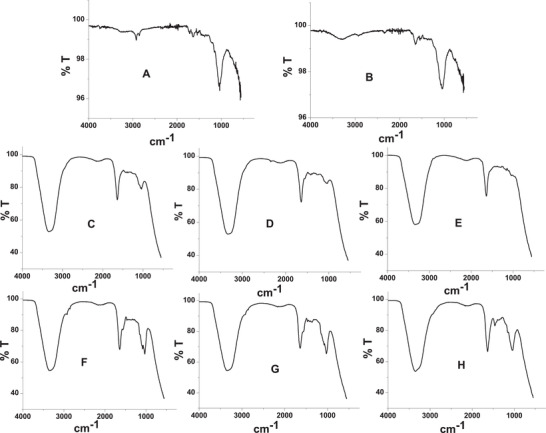
FT‐IR spectra of A) native rice bran (RB), B) pre‐treated RB, C) Type A pH 6, D) Type A pH 8, E) Type A pH 10, F) Type B pH 6, G) Type B pH 8 and H), Type A pH 10, respectively.

The FTIR spectra of the raw RB, pretreated RB (treated in hexane), and chemically modified RB adhesives (Type A and B) are shown in **Figure** **4**. As seen in Figure 4, sharp vibrational frequency peaks appeared at around 1033 and 1046 cm^–1^ for the native RB powder and pretreated RB, respectively, that are attributed to the C—OH stretching vibration of the starch molecules.^[^ ^41^ ^]^ The peaks appearing at 1033 and 1046 cm^–1^ are the characteristics peaks of crystalline and/or orderly structure of the starch molecules of the RB powder.^[^ ^42^ ^]^ The orderly and/or crystalline structure of the starch molecules did not change to amorphous though the RB powder was treated by hexane because of appearance of a sharp peak at 1046 cm^–1^ for the pretreated RB powder (Figure 4B).

The raw RB powder also showed the characteristic vibrational peaks of C—H stretching at around 2922 cm^–1^ (sp^2^ C—H stretching) and at a lower frequency (2865 cm^–1^) for sp^3^ C—H stretching (Figure 4A). On the other hand, the pretreated RB powder showed the C—H vibrational frequency peaks (for both sp^2^ and sp^3^ C—H stretching) with very low intensities at the same frequency regions as shown by the RB powder, whereas a broad peak appeared at 3282 cm^–1^ for the pretreated RB (Figure 4B). The broad peak at 3282 cm^–1^ indicates the presence of hydroxyl group (O—H) of the starch molecules of the RB. The peak for O—H at lower frequency (3282 cm^–1^) can be explained owing to formation of intermolecular hydrogen bonding of the starch molecules through the O—H groups. It has been speculated that defatting the raw RB powder by hexane facilitates the formation of intermolecular hydrogen bonding of the starch molecules by using the O—H groups.

On the other hand, both the chemically modified RB adhesives showed high intense peak at around 3340 cm^–1^ (Figure 4C–H). The peak appearing at 3340 cm^–1^ could be attributed to both the O—H and N—H groups. In Type B adhesive, a mixture of urea (5.7%) and formaldehyde (10.8%) was used for chemical modification, whereas neither urea nor formaldehyde was used in preparation of Type A adhesive. Therefore, it can be concluded that the peak appearing at 3340 cm^–1^ indicates the presence of O—H group in the adhesives. As mentioned in the preceding sections, the pretreated RB showed a broad peak with very low intensity at 3282 cm^–1^ which was attributed to hydrogen‐bonded O—H group of the starch molecules. The use of higher content of water for the preparation of chemically modified RB adhesives was responsible to show high intense peak at around 3340 cm^–1^ which was attributed to the O—H group of the bound water molecules in the starch network. Whether the presence of water molecules in the chemically modified adhesives was responsible or not for appearing the peak at 3340 cm^–1^, the prepared adhesives (both Types A and B) were kept in a desiccator for 24 h and then IR spectra were recorded (spectra are not shown). No spectral change was observed before and after desiccating the samples. The water molecules probably bound strongly to the glucose network of the pretreated RB, thus, desiccating was not an effective method to remove the strongly bound water molecules.

Both the chemically modified adhesives (Types A and B) exhibited the vibrational frequency peak at around 1640 cm^–1^ in the pH range from 6 to 10, which was attributed to the stretching vibration of —C=O group in amide. This suggests the presence of protein in the adhesives.^[^ ^42^ ^]^ Type A adhesive showed only a single vibrational peak with a very low intensity at around 1054 cm^–1^ at pH 6, and the intensity decreased with increasing the pH and almost disappeared at pH 10. On the other hand, type B adhesive prepared at pH 6 showed two distinct vibrational frequency peaks at 1078 and 1027 cm^–1^ that were attributed to C—O—C (stretching) and C—OH (stretching), respectively. With increasing pH (8, 10), the peak at 1078 cm^–1^ tended to disappear and finally disappeared at pH 10 and showed a single broad peak at 1055 cm^–1^. The peak appearing at 1462 cm^–1^ for pH 10 was attributed to —CH_2_— bending.

As mentioned above, the starch molecules in the native RB and pretreated RB samples are mostly crystalline by observing the characteristic peaks appeared at 1033 and 1046 cm^–1^, respectively (Figure 4A,B).^[^ ^42^ ^]^ Type A adhesive prepared from the mixing of native RB with water, 1% KMnO_4_ and PVA, showed only a peak with a very low intensity that appeared at 1054 cm^–1^ at pH 6 and disappeared with increasing the pH such as 8 and 10. This result confirms that the permanganate‐based chemical treatment disrupts the crystalline/ordered structure of the starch molecules and converts into amorphous at acidic pH 6.^[^ ^42^ ^]^ The very low intensity of the peak appeared at 1054 cm^–1^ at pH 6 and that almost disappeared at pH 8 or 10 suggested that the permanganate‐based treatment is rather a strong chemical method that causes severe chemical modification of the starch molecules. On the other hand, Type B adhesive prepared from the native RB along with urea and formaldehyde has shown the characteristic peak along with another peak appeared at 1027 and 1078 cm^–1^ for amorphous starch molecules at acidic pH 6.^[^ ^42^ ^]^ This result suggests that the urea‐formaldehyde chemical method is relatively mild that mostly disrupts the crystalline structure of the starch molecules into amorphous in acidic condition (pH 6); however, this method does not cause significant chemical change of the starch molecules. Therefore, it may conclude that permanganate‐based chemical method is rather strong than the urea‐formaldehyde method for chemical modification of the starch molecules.

### 3.7 Shear Strength

**Figure 5:**
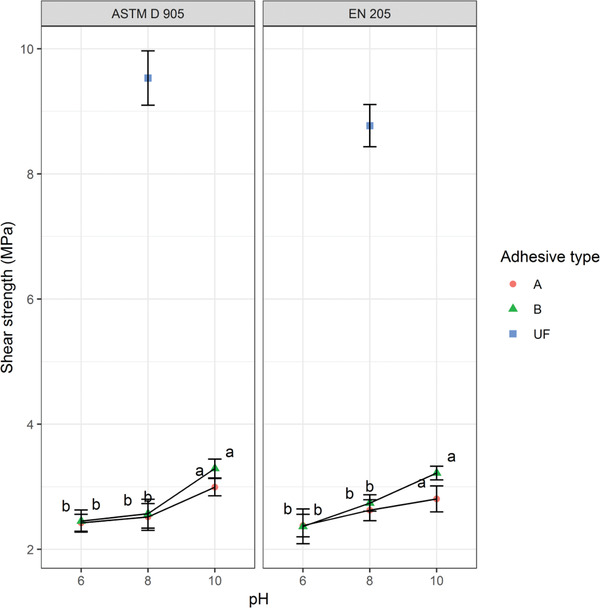
Average shear strength of adhesives tested in the study.

FTIR and DSC techniques are not capable to provide clues about chain extension from crosslinking reactions, therefore the data obtained from these two techniques are not directly informative about the mechanical properties developed in the matrices.^[^ ^43^ ^]^ However, shear test can provide the clue about the mechanical properties developed in the adhesives. **Figure** **5** shows the effects of pH and chemical treatment methods on the shear strengths of the RB adhesives measured according to ASTM D 905 and EN 205 standards. According to ASTM standard, the shear strengths for Type A and B adhesives increased significantly (ANOVA, *F*_2,36_ = 157.0109, *P* < 0.05) from 2.42 to 2.99 MPa and from 2.45 to 3.29 MPa for changing pH from 6 to 10, respectively. Similar increasing trend of the shear strength measured by EN standard as a function of pH was also found. According to EN standard, the shear strengths increased from 2.38 to 2.82 MPa for Type A and from 2.37 to 3.21 MPa for Type B with increasing of pH from 6 to 10, respectively. Meanwhile, Wang et al.^[^ ^9^ ^]^ and Wang et al.^[^ ^22^ ^]^ reported that the shear strengths of the RB adhesives ranged from 0.9 to 1.16 MPa and 1.9 to 2.3 MPa, respectively,^[^ ^9^, ^22^ ^]^ which were relatively lower than those of the present study. Eventually no effect of the treatment methods on the shear strengths of the RB adhesives was observed.

As mentioned above, the defatted RB contains mainly starch along with 12–16% proteins.^[^ ^44^, ^45^ ^]^ The presence of water in the RB facilitates the formation of gelatinous matrix at 70 °C which enhances the adhesive properties of the RB.^[^ ^46^, ^47^ ^]^ The protein consists of mainly 37% water‐soluble, 31% salt‐soluble, and 27% alkali‐soluble proteins; therefore, most of the proteins are released to aqua solution phase when treated at alkaline pH (8–10).^[^ ^45^ ^]^ The proteins in solution phase, thus, take part in crosslinking network of the gelatinous starch resulting in enhancing adhesive strengths of the RB.^[^ ^46^, ^47^, ^48^, ^49^ ^]^

In the presence of water, starch is gelatinized at the absolute gelatinization temperature to develop its adhesive properties, and above the gelatinization temperature, protein also increases its strength properties.^[^ ^46^, ^47^ ^]^ The pH–chemical treatments interaction also influenced the increase of the RB adhesive strength (Figure 5). Protein solubility enhances the strength properties of RB adhesive and its solubility increases at pH ≥ 8.^[^ ^48^ ^]^ Though the resulting shear strength was much lower than the commercial grade UF resin, it was better in contrast to the previous studies of RB adhesive such as 1.9–2.3 MPa^[^ ^20^ ^]^ and 0.9–1.16 MPa.^[^ ^9^ ^]^

### 3.8 Variability in Shear Strength Values

**Figure 6:**
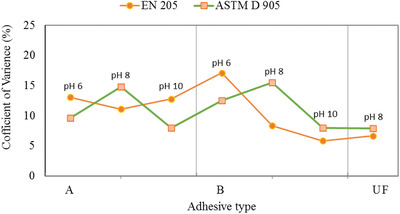
Coefficient of variance (CV) of shear strength results according to type of adhesive and testing procedures.

Coefficient of variance (CV) for a set of experimental data is an important factor to compare the variability or stability of different testing methods. The CV is usually expressed as percentage, and it is the ratio of standard deviation (σ) to the mean value (*µ*) for an essay. The CV is widely used in analytical science to express the precision and repeatability for a set of experimental data. Low value of CV indicates high stability with less variability of the applied method that provides highly precise experimental data and vice versa. The CV values obtained from a set of shear strengths measured by ASTM D 905 and EN 205 methods are shown in **Figure** **6**.

Figure 6 indicates that the average CV value measured by the ASTM D 905 method was 13.08% with a range from 7.91% to 15.49% whereas that by EN 205 was 12.49% with a range from 6.54% to 20.35%. EN method showed a significant fluctuation among the data, even though the average percentage of the CV value by EN method was relatively smaller compared to that by ASTM. Therefore, it may be concluded that both the methods are valid to measure the shear strengths of the prepared RB adhesives.

### 3.9 Physical and Mechanical Properties of the Produced Particleboard

**Figure 7:**
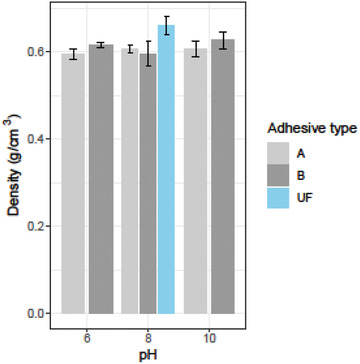
Density of the fabricated particleboards using adhesive type A, adhesive type B, and UF at different pH.

The density of the particleboards made with both types of RB adhesives (Type A and Type B) was lower than that of UF‐based particleboard (**Figure** **7**). The density ranged between 0.60 and 0.61 g cm^−3^ for Type A adhesive based particleboards at different pH level; however, it was 0.60–0.63 g cm^−3^ for Type B adhesive based particleboards at different pH. The effect of pH on particleboards’ density was not significant for both types of adhesive, and the types of adhesives were not significant as well. However, the higher pH enhanced board density for both types of RB adhesives except particleboards made with Type B adhesive at pH 8. The different chemical treatments for the preparation of adhesives^[^ ^50^ ^]^ and applied different pH values for manufacturing particleboards resulted in variation in particleboards’ density. On the other hand, wood particle has free hydroxyl (OH) group,^[^ ^51^, ^52^, ^53^ ^]^ and the higher pH can trigger up the number of OH group in the matrix, which can make more OH bonds with particles. Thus, the higher density particleboards may be possible for RB adhesives made with higher pH. The particleboards made with modified wheat and palm oil starch were 0.61 g cm^−3^ in previous study.^[^ ^54^ ^]^ It was more or less similar to the density of boards made with RB adhesives in this study.

**Figure 8:**
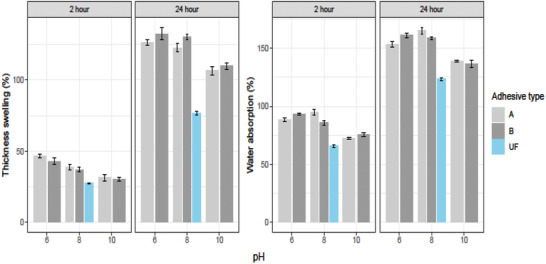
Thickness, swelling, and water absorption after 2 and 24 h of the fabricated particleboards using adhesive type A, adhesive type B, and UF at different pH

The measured WA and TS of the particleboards at 2 and 24 h are presented in **Figure** **8**. The figure includes the WA and TS of all the particleboards made from UF and RB‐baed adhesives. Meanwhile, the particleboards made with RB adhesives at pH 10 had lower value of WA and TS for 2 and 24 h immersion in water. The WA was lower 18.0% and 9.8% for 2 and 24 h immersion in water, respectively, for Type A adhesive when pH increased from 6 to 10. For Type B adhesive, the increment of pH from 6 to 10 lowered the WA 19.0% and 14.9% for 2 and 24 h immersion in water, respectively. The TS was decreased by 32.9% and 15.8% with increasing the pH from 6 to 10 for 2 and 24 h immersion in water, respectively, for Type B adhesive. Again, the increment of pH from 6 to 10 lowered the TS by 32.8% and 18.5% for 2 and 24 h of soaking in water, respectively, for Type B adhesive. However, OH group free UF resin is nondegradable in water^[^ ^55^ ^]^ and thus, resulting in lower WA consequently lower TS compared to RB adhesive. On the other hand, the higher pH means more OH group bond with wood particle. The excessive OH groups may work as inhibitor of water owing to the cohesive force among the particles.^[^ ^56^ ^]^ Again, the low density indicates more void in the matrix of particleboards leading to uptake of more water, which results in more WA and TS.^[^ ^57^ ^]^ Therefore, the particleboards prepared by RB adhesive at higher pH (pH 10) showed lower values of WA and TS. Epichlorohydrin‐modified oil palm starch based particleboards showed WA of 114% for 2 h and 123% 24 h and TS of 43% for 2 h and 54% for 24 h in previous study.^[^ ^2^ ^]^ The WA and TS except TS for 24 h were lower for particleboards prepared from RB‐based adhesives than those of previous findings. In this study, the performance of Type B adhesive was comparatively better than Type A adhesive.

The values of WA and TS for the fabricated particleboards were ranging from 73 to 165% and 75 to 161% for Type A and 31–127% and 30–133% for Type B, respectively (**Table** **4**). Basta et al. (2016) investigated the agro‐based biocomposites where they used rice straw (RS) as a base matrix and starch/polyvinyl as an adhesive.^[^ ^64^ ^]^ They found the TS values ranging from 40 to 70% for the fabricated particleboards (Table 4).^[^ ^64^ ^]^ However, in other studies, the TS values for the particleboards fabricated by using starch/UF‐RS and RB/UF‐bagasse were found to be ranging from only 12 to 18% and 4.1 to 34.7%, respectively (Table 4).^[^ ^65^, ^66^, ^67^ ^]^ Nicolao et al. (2020) also found only 22–30% of TS for the particleboards prepared from rice husk‐jute stick where soy protein was used as an adhesive (Table 4).^[^ ^68^ ^]^ They also measured density and WA of the particleboards and found to be 0.80–0.82 g cm^−3^ and 45–77%, respectively (Table 4).^[^ ^68^ ^]^ In another study, WA value was found to be 30–50% for the particleboards fabricated from bagasse using starch/UF as an adhesive (Table 4).^[^ ^69^ ^]^ As mentioned above, we found high values of WA and TS for the particleboards prepared from wooden particles and RB‐based adhesives and the values were 73–165% and 31–127% for Type A and that for Type B were 75–161% and 30–133%, respectively (Table 4). Defatting of the RB may cause adsorption and/or absorption of moisture that enhances the values of WA and TS for the particleboards fabricated from Types A and B adhesives.

**Table 4:** Comparative results for formaldehyde free or low toxic adhesive based composites

Type of adhesive	Type of composite	Physical properties			Mechanical properties				Reference
		Density [g cm^−3^]	WA [%]	TS [%]	MOE [MPa]	MOR [MPa]	Tensile strength [MPa]	Hardness [MPa]	
Type A (RB/polyvinyl alcohol)	Particleboards	0.60–0.61	73–165	31–127	1198–1896	7.24–10.61	2.99–3.96	0.51–0.68	Current study
Type B (RB/UF)		0.60–0.63	75–161	30–133	1207–1944	7.34–10.69	3.02–4.13	0.49–0.71	
Starch/polyvinyl alcohol	Rice straw‐based particleboards	nd	nd	40–70	nd	14–25	nd	nd	^[^ ^64^ ^]^
Starch/UF	Rice straw‐based particleboards	nd	nd	12–18	nd	10–16	nd	nd	^[^ ^65^, ^66^ ^]^
RB/UF	Bagasse‐based particleboard	nd	nd	4.1–34.7	2505–3898	15.6–29.6	nd	nd	^[^ ^66^, ^67^ ^]^
Soy protein	Rice husk and jute‐based particleboards	0.81–0.82	45–77	22–30	1725–2750	11.0–16.5	4.7–12.5	nd	^[^ ^68^ ^]^
Starch/UF	Bagasse‐based particleboard	nd	30–50	Nd	3000–5000	25–27	nd	nd	^[^ ^69^ ^]^

*Note*: UF, RB, WA, TS, MOE, and MOR refer to urea formaldehyde, water absorption, thickness swelling, modulus of elasticity, and modulus of rupture, respectively. “nd” indicates “not done” by the respective authors.

**Figure 9:**
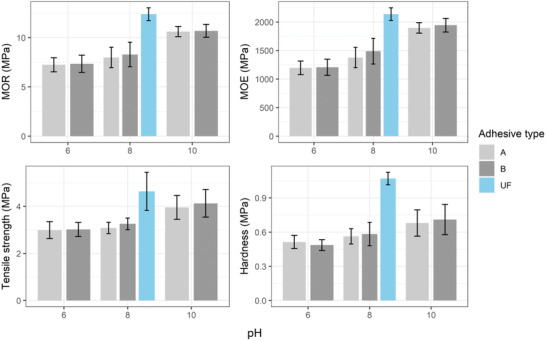
MOR, MOE, tensile strength, and hardness of the fabricated particleboards using adhesive type A, adhesive type B, and UF at different pH.

The determined mechanical properties, i.e., MOE, MOR, tensile strength, and hardness are shown in **Figure** **9**. The UF‐based particleboards showed higher value of mechanical properties compared to that of RB‐based particleboards (Figure 9). The mechanical properties of particleboards increased with increasing pH for both types of RB adhesives (Figure 9). The MOE, MOR, tensile strength, and hardness increased by 58.3, 46.5, 32.2, and 32.5%, respectively, for increasing pH from 6 to 10 for Type A adhesive. In case of Type B adhesive, the increment was 61.1, 45.5, 36.8, and 45.9% for MOE, MOR, tensile strength, and hardness, respectively. The mechanical properties are strongly correlated with density of particleboards.^[^ ^57^ ^]^ The density of particleboards was higher for UF‐based particleboards, and the particleboards made with Type B adhesive at pH 10 had higher density among RB‐based adhesives. Therefore, the mechanical properties of particleboards made with Type B adhesive at pH 10 were higher among RB‐based adhesives. Starch‐based adhesives enhance the filler fraction, crosslinking network, and interfacial adhesion in the composite matrices.^[^ ^59^, ^60^ ^]^ The higher number of OH^–^ groups may enhance the bonding ability between adhesive and wood particles. Accordingly, the mechanical properties were higher for the particleboards made with Type B‐based RB adhesives treated at pH 10. The particleboards made with RB‐based adhesives followed the ASTM standard D1037‐99 requirements for all the tested mechanical properties.^[^ ^61^ ^]^ Starch, on the other hand, can provide better shear stability leading to stable mechanical properties.^[^ ^62^, ^63^ ^]^ A comparative result of formaldehyde free or low toxic adhesive based composites of previous studies along with the present study has been shown in Table 4.

As seen from Table 4, the values of MOR for the particleboards made with rice straw using starch/polyvinyl and starch/UF as adhesives were 14–25 and 10–16 MPa, respectively (Table 4).^[^ ^64^, ^65^, ^66^ ^]^ The MOR value for the bagasse/RB/UF‐based particleboards was 15.6–29.6 MPa (Table 4).^[^ ^66^, ^67^ ^]^ A comparable value of the MOR (25–27 MPa) was also found for the bagasse‐based particleboards where starch/UF was used as an adhesive.^[^ ^68^ ^]^ However, Basta et al. (2013) reported relatively low values of the MOR (11.0–16.5 MPa) for the particleboards prepared from rice husk‐jute stick as base materials and soy protein as an adhesive (Table 4).^[^ ^69^ ^]^ In most of the cases, the presence of UF in the adhesives enhances the mechanical properties of the particleboards (Table 4).^[^ ^65^, ^66^, ^67^, ^68^ ^]^ The values of the MOE for the particleboards fabricated from bagasse were found to be 2505–3898 and 3000–5000 MPa for RB/UF and starch/UF, respectively;^[^ ^66^, ^67^, ^68^ ^]^ however, in this study, we found only 1207–1944 MPa of MOE for the particleboards prepared from wood particles and RB/UF adhesive (Table 4). It is expected to obtain low values of the mechanical properties for the particleboards made by UF‐free adhesives observed in this study for Type A adhesive and in another study investigated by Basta et al. (2016) (Table 4).^[^ ^69^ ^]^ These results suggest that the presence of UF in the adhesives is not only the reason for the enhancement of the mechanical properties of the fabricated particleboards.

### 3.10 Formaldehyde Emission from Particleboards

The results obtained from the formaldehyde emission analysis showed that particleboards fabricated from the adhesive Type A, B, and UF were 0, 0.0011, and 0.0054 ppm, respectively, whereas the allowable limit of formaldehyde in home is 0.03 ppm recommended by USCPSC.^[^ ^70^ ^]^ From this observation, it is clear that there was no formaldehyde emission from the particleboard fabricated from Type A adhesive as formaldehyde was not used to formulate the adhesive. Whereas the prepared particleboard from Type B adhesive reduces the formaldehyde emission by 80% compared to the commercial UF fabricated particleboard. In the previous study, the range of formaldehyde emission was 0.0042–0.0068 ppm for the particleboards made from formaldehyde‐based adhesive.^[^ ^71^ ^]^ Moreover, the emission data obtained in the study were much lower than the emission found from particleboards fabricated from starch and nontoxic materials with UF.^[^ ^69^, ^72^ ^]^ Therefore, RB‐based adhesive might be a promising adhesive to reduce the formaldehyde emission from the wood‐based industries.

## 4 Conclusion

In this study, a renewable and environmentally friendly chemically modified RB adhesive was obtained through polycondensation reactions between RB and chemicals. Chemical treatment in alkaline condition had proven as an effective method to prepare the RB adhesives with better strength. The enhanced strength of the RB adhesives was confirmed through studying the IR spectra of various functional groups of the raw and chemically treated RB adhesives. The chemically prepared RB adhesives exhibited better strength at pH 10. The physical and mechanical properties of RB adhesives based particleboards were also higher when there was higher pH. Though both the adhesives (Types A and B) satisfied the minimum requirements as a quality adhesive, their properties were a little bit lower than that of the commercial UF resin. Between the Type A and Type B adhesives, Type B was comparatively better than Type A. These issues merit further research to improve the bonding strength properties of the adhesives.

## Conflict of Interest

The authors declare no conflict of interest.
